# Reduced Plasma Dopamine-β-Hydroxylase Activity Is Associated With the Severity of Bipolar Disorder: A Pilot Study

**DOI:** 10.3389/fpsyt.2021.566091

**Published:** 2021-04-28

**Authors:** Zuoli Sun, Qijing Bo, Zhen Mao, Feng Li, Fan He, Christine Pao, Wenbiao Li, Yi He, Xin Ma, Chuanyue Wang

**Affiliations:** ^1^The National Clinical Research Center for Mental Disorders and Beijing Key Laboratory of Mental Disorders and Beijing Institute for Brain Disorders Center of Schizophrenia, Beijing Anding Hospital, Capital Medical University, Beijing, China; ^2^Advanced Innovation Center for Human Brain Protection, Capital Medical University, Beijing, China; ^3^Department of Psychiatry, University of North Carolina at Chapel Hill, Chapel Hill, NC, United States

**Keywords:** dopamine-β-hydroxylase, bipolar disorder, major depressive disorder, mood disorder, cognitive function

## Abstract

Dopamine-β-hydroxylase (DβH) is an enzyme converting dopamine to norepinephrine, a key neurotransmitter in mood disorders, such as major depressive disorder (MDD) and bipolar disorder (BD). Due to overlapping symptomology of unipolar and bipolar depression, the present study attempted to explorer if the plasma DβH activity could discriminate the depressive episodes of BD from MDD. The aim of this study was to compare the plasma DβH activity among MDD patients (*n* = 104), BD patients (*n* = 101), and healthy controls (*n* = 160). Clinical characteristics and cognitive function were assessed using the Young Mania Rating Scale (YMRS), Hamilton Depression Scale (HAM-D), Hamilton Anxiety Scale (HAM-A), Patient Health Questionnaire-9 (PHQ-9), and Repeatable Battery for the Assessment of Neuropsychological Status (RBANS). Our data showed a lower plasma DβH activity in patients with BD, not MDD, than that in controls. For the BD patients, the plasma DβH activities were negatively correlated with HAM-D scores and HAM-A scores. However, there was no significant correlation between plasma DβH activity and severity of depressive symptoms in MDD patients. No significant correlation between DβH activities and cognitive assessments neither in BD nor in MDD patients. The present study provides evidence that BD is associated with decreased circulating DβH activity.

## Introduction

Bipolar disorder (BD) is a complex and chronic psychiatric disease, with a prevalence to range from 0.5 to 5% in community-based samples (1, 2). BD is a disabling disease due to its early onset, severity and chronic nature although relatively rare (3). The important character of BD is the alternative episodes of mania and depression. However, major depressive disorder (MDD) and BD at depressive phase are similar in clinical manifestations, their overlapping symptomology makes difficult to differentiate BD at depressive phase from MDD (4). More importantly, the use of antidepressants in BD patients have limited efficacy even might increase the possible switch to manic episodes (5). Therefore, it is important to find biomarker to differentiate BD at depressive phase from MDD, thereby to improve the therapeutic effect on mood disorders.

Unfortunately, the underlying mechanisms of BD and MDD have not yet been fully elucidated, while the monoaminergic theory has been regarded as the main cause of both MDD and BD (6–8). According to the monoamine hypothesis, monoamine neurotransmitters, including serotonin (5-HT), dopamine (DA), and norepinephrine (NE), have always been the key aspects in mood disorders. Although previous genetic, biological and pharmacological studies confirmed the important role of 5-HT in MDD (7, 9), accumulating evidence suggested the contribution of dysregulated DA and NE transmission on both MDD and BD (10–12). DA and NE play an important role not only in cognitive function, but also in emotion regulation (13, 14). In general, depression was associated with reduced activity in the dopaminergic signaling system (15), whereas mania was associated with increased dopaminergic signaling (16). For example, depressive symptoms have been linked with hypodopaminergic transmission in the reward system (17), and dopaminergic stimulant agents augment the efficacy of antidepressants (18). On the other hand, hyperdopaminergic mice showed mania-like behaviors (19), and changed levels of 3-methoxy-4-hydroxyphenylglycol (MHPG, metabolites of NE) and homovanillic acid (HVA, metabolites of DA) were found in BD patients compared to controls (10, 20). Thus, the different changes of DA and NE transmission may contribute to explore the mechanism of MDD and BD.

Dopamine β-hydroxylase (DβH) is the key regulatory enzyme required to synthesize NE from DA (21), and it is also important to maintain the brain DA/NE balance. DβH is located in both central (catecholamine vesicles) and peripheral systems (sympathetic nerves and adrenal medulla) (22, 23). Since DβH can be released from catecholamine vesicles, the protein can be determined in the plasma or serum (21). Previous studies with mice deficient in DβH demonstrated the role of DβH in mood disorders. The birth rate of DBH gene knockout mice was much lower than wild-type mice, and the surviving mice almost died in the first week of life (24), suggesting the important role of DβH in development and survival. Considering the important effect of NE and DA in cognition, several evidences indicated that DβH might play a role in the cognitive defects in mental disorders (25–29), although DβH-deficient patients failed to display neurocognitive impairment (30, 31).

Abundant evidence has shown that low serum/plasma DβH activity might be a risk factor for mental illness (21, 25, 27, 32). Furthermore, the genetic, biological and pharmacological studies indicated the important role of DβH in BD and MDD. The *DBH* gene variants were proved to join in predicting individual differences in social and affective processing (33). Ates et al. indicated the mutated *DBH* gene increased the risk of BD (34). In addition, the serum DβH activity was significant decreased in BD patients in depressive state compared with MDD patients (35), while it was lower in MDD patients than that in healthy controls (36–40). Interestingly, compared with drug-naive BD patients, serum DβH activity was higher in lithium-treated BD patients (41). However, other studies showed the contrary results (42, 43). Animal studies indicated stress increased expression of DβH mRNA and protein in mood-related areas in the brain (44, 45). The contrary studies suggested the complex role of DβH in patients with mood disorders, even between MDD patients and BD patients in depressive state.

Due to overlapping symptomology of unipolar and bipolar depression, the present study attempted to explorer if the plasma DβH activity could discriminate the depressive episodes of BD from MDD. The aim of this study was to investigate whether plasma DβH activity differs between: (i) BD patients and healthy controls (HCs), (ii) MDD patients and HCs, (iii) BD patients and MDD patients. The plasma DβH activity was tested in individuals with three groups: BD patients, MDD patients and HCs. Furthermore, the associations of DβH activity and phenotypes of patients were also assessed in this study.

## Materials and Methods

### Subjects

Subjects were recruited from Beijing Anding Hospital (China) from September 2014 to September 2016. The ethics committee of Beijing Anding Hospital approved the research. All of the individuals provided written informed consent to participate in the present study after fully explaining the purpose and procedure. Male and female patients aged 16 to 60 years who met the Diagnostic and Statistical Manual of Mental Disorders, Fourth Edition (DSM-IV) criteria for MDD, or BD were recruited. All patients were screened using the Structured Clinical Interview for DSM-IV Axis I disorders-Patient Edition (SCID) by experienced psychiatrists. In the present study, all the patients with MDD were undergone depressive episodes, while the BD patients also with a current depressive episode were recruited.

The inclusion criteria of all patients were as follows: (1) aged 16 to 60 years; (2) formal education ≥9 years; (3) total scores of Young Mania Rating Scale (YMRS) ≤ 6. The exclusion criteria of all patients were as follows: (1) comorbidity with other psychiatric disorders, such as schizophrenia; (2) electric convulsive therapy in recent 3 months; (3) were or had a history of substance dependence; (4) severe suicidal tendencies; (5) severe physical diseases, such as neurological diseases, cardiovascular disease, hepatic or renal diseases; (6) current pregnancy or breastfeeding.

The present study recruited gender-matched HC individuals with no history of psychosis or cognitive impairment. HCs underwent a psychiatric interview by experienced clinicians using the SCID to exclude psychiatric disorders. HCs were excluded when encounter the following situations: (1) had any lifetime DSM-IV psychiatric disorder; (2) had severe physical diseases, such as neurological diseases, cardiovascular disease, hepatic or renal diseases; (3) had a family history of psychiatric diseases; (4) were or had a history of substance dependence; (5) were current pregnancy or breastfeeding.

### Clinical Assessments

Clinical assessments were administrated by experienced psychiatrists, who were blinded to participants. The patients were assessed with several clinical scales, including the Young Mania Rating Scale (YMRS) (46), Hamilton Depression Scale (HAM-D) (47), Hamilton Anxiety Scale (HAM-A) (47), and Patient Health Questionnaire-9 (PHQ-9) (48). In addition, the cognitive function of each participant was assessed with Wechsler Adult Intelligence Scale (brief form), Stroop's color-word test (49), and Repeatable Battery for the Assessment of Neuropsychological Status (RBANS) (50). Five aspects of the RBANS were evaluated, including attention, speech, visual span, immediate memory, and delayed memory.

### Plasma DβH Activity Assay

Peripheral blood from each individual was collected in this study. Plasma was harvested and stored at −80 °C until they were used for the detection of DβH activity. The DβH activity was detected with the method reported from our laboratory (25), which was based on the enzymatic conversion of tyramine (substrate) to octopamine by DβH. Briefly, the reaction mixture (100 μL) was composed of 1 M CH3COONa buffer (pH = 5.0), 0.2 M sodium fumarate, 0.2 M ascorbic acid, 1,500 U catalase, 0.2 M tyramine hydrochloride, 0.02 M pargyline, 0.2 M N-ethylmaleimide, pure water and plasma (5 μL). After being incubated at 37°C for 1 h, the reaction was stopped by adding 20% HClO4 (20 μL). The supernatant was transferred to high performance liquid chromatography (HPLC) system after centrifugation (2,000 rpm) for 10 min. Octopamine concentration was measured by column-switching, reverse phase HPLC system (U3000, Thermo Fisher Scientific, Waltham, MA, USA), with electrochemical detection. Electrodes 1 and 2 of the cell were set at +700 and −320 mV, respectively. Synephrine was used as internal standard. Octopamine and synephrine were separated by a 5 μm particle size reversed-phase SB-C18 analytical column (2.1 × 150 mm) obtained from Agilent Technologies (Santa Clara, CA, USA). The mobile phase was composed of 50.9 mM potassium acetate, 14.3 mM citric acid, 1.38 mM 1-octanesulfonic acid, and 9.5% methanol (v/v). All chemicals were purchased from Sigma-Aldrich (St. Louis, MO, USA).

### Statistical Analysis

The data were analyzed using SPSS with version 20.0 (SPSS Inc., Chicago, IL, USA). Demographic and clinical characteristics were compared among three groups using χ^2^-tests or one-way analysis of variance (ANOVA). Consideration of the significant difference in age, the covariance analysis was used to compare the continuous variables, including plasma DβH activities among groups with age as a covariate. On the other hand, the multivariate logistic regression analysis was used to evaluate the associations between discontinuous variable, including sex, family history, current state of illness, and mood disorders susceptibility with adjustment for age. Two-way ANOVA was used to evaluate the interaction effects between groups and gender, or compare the plasma DβH activity in patients with different episodes. The comparison of plasma DβH activity in patients with different kinds of drugs was also used with two-way ANOVA. In order to minimize the bias of demographic data on the analysis, the partial correlation was used to analyze the association of DβH activity and clinical and cognitive assessments, with age, education, duration of illness and first onset age as covariates. The level of significance was set at *p* < 0.05. However, the corrected *p-*value was set at 0.0167 considering the multiple testing of cognitive functions of the patients in the partial correlation analysis.

## Results

### Demographic and Clinical Characteristics

A total of 389 individuals were initially screened for this study, and 24 (14 in MDD group, six in BD group, and four in control group) were excluded due to the declining to participate, not drawing blood or not meeting the inclusion criteria. Ultimately, the study population comprised of 104 MDD patients, 101 BD patients and 160 HCs. Although more than 90% patients completed all the clinical and cognitive assessments, only 116 HCs completed all the clinical assessments. The comparison of demographics and clinical characters among these three groups is shown in Table 1. Notably, there was a significant difference in age among groups (*F* = 6.795, *p* = 0.001). Significant differences in the YMRS, HAM-D, and HAM-A total scores were found among three groups. More concretely, the HAM-D, HAM-A, and PHQ-9 scores were significantly increased in MDD or BD patients compared to HCs. However, the YMRS scores were higher in BD patients than MDD patients or HCs. Furthermore, these differences still exist after covariance analysis controlling for age (Table 1). In addition, according to the assessments of intelligence quotient (IQ), RBANS, and Stroop's tests, the MDD or BD patients showed significant deficits in cognitive function.

**Table 1:** Demographics and other characteristics in three groups.

	**HC**	**MDD**	**BD**	**F**	***p***	***p_**Covariance**_***
**General characteristics**						
*n*	160	104	101			
Gender (male/female)	80/80	54/50	62/39	3.413^a^	0.182	0.121^c^
Age (years)	30.42 ± 9.21	34.77 ± 11.13	30.48 ± 10.40	6.795	0.001	NA
Education (years)	13.77 ± 3.17	13.06 ± 3.07	13.09 ± 3.13	1.826	0.163	0.200
Duration of Illness (months)	NA	67.75 ± 69.83	86.90 ± 73.72	3.647	0.058	0.002
First onset age	NA	29.59 ± 11.25	23.49 ± 9.25	17.933	<0.001	0.002
Family history (yes)	NA	26	32	1.101^a^	0.294	0.238^c^
Type of first episode (depression/mania)	NA	NA	75/26			
Current state (remission/episodes)	NA	48/56	58/43	2.84^a^	0.096	0.067^c^
**Medication (treated/untreated)**						
Mood stabilizers	NA	3/101	71/30			
Antipsychotics	NA	20/84	63/38			
Antidepressants	NA	79/25	27/74			
**Symptom assessment**						
YMRS	1.13 ± 2.13	0.88 ± 1.84	3.27 ± 6.02	11.358	<0.001	<0.001
HAM-D	0.40 ± 1.13	11.34 ± 8.95	8.92 ± 9.06	53.776	<0.001	<0.001
HAM-A	0.42 ± 1.09	10.82 ± 9.46	7.94 ± 8.40	47.699	<0.001	<0.001
PHQ-9	3.36 ± 6.02	10.39 ± 7.55	7.57 ± 7.38	17.634	<0.001	<0.001
**Cognitive assessment**						
**IQ**	108.42 ± 17.46	100.69 ± 16.32	98.74 ± 13.11	9.715	<0.001	<0.001
**RBANS**						
Attention	108.16 ± 14.54	102.05 ± 18.57	101.53 ± 14.11	4.878	0.008	0.001
Speech	98.14 ± 18.65	90.42 ± 14.56	85.37 ± 14.63	14.857	<0.001	<0.001
Visual span	100.65 ± 17.76	96.98 ± 17.09	98.18 ± 15.70	1.127	0.325	0.229
Immediate memory	91.14 ± 18.06	83.42 ± 20.09	80.22 ± 17.09	8.431	<0.001	<0.001
Delayed memory	93.85 ± 13.24	85.59 ± 19.15	83.58 ± 15.16	10.383	<0.001	<0.001
Total scores	97.27 ± 15.54	88.92 ± 17.64	85.82 ± 11.34	14.523	<0.001	<0.001
**Stroop**						
Single word time	15.49 ± 5.02	17.24 ± 6.12	17.76 ± 5.42	4.139	0.017	0.001
Monochromatic time	20.17 ± 6.28	24.03 ± 9.81	26.74 ± 11.12	11.298	<0.001	<0.001
Double words time	19.20 ± 8.24	21.30 ± 10.09	23.55 ± 10.61	4.558	0.011	<0.001
Double color time	36.64 ± 12.11	41.70 ± 15.13	44.79 ± 16.56	7.043	0.001	<0.001

*Values represent mean ± S.D*.

a *means χ^2^ test*.

c *means multivariate logistic regression analysis*.

*HC, Healthy controls; MDD, Major depression disorder; BD, Bipolar disorder; YMRS, Young Mania Rating Scale; HAM-D, Hamilton Depression Scale; HAM-A, Hamilton Anxiety Scale; PHQ-9, Patient Health Questionnaire-9; IQ, Intelligence quotient; RBANS, Repeatable Battery for the Assessment of Neuropsychological Status*.

### Plasma DβH Activity

**Figure 1:**
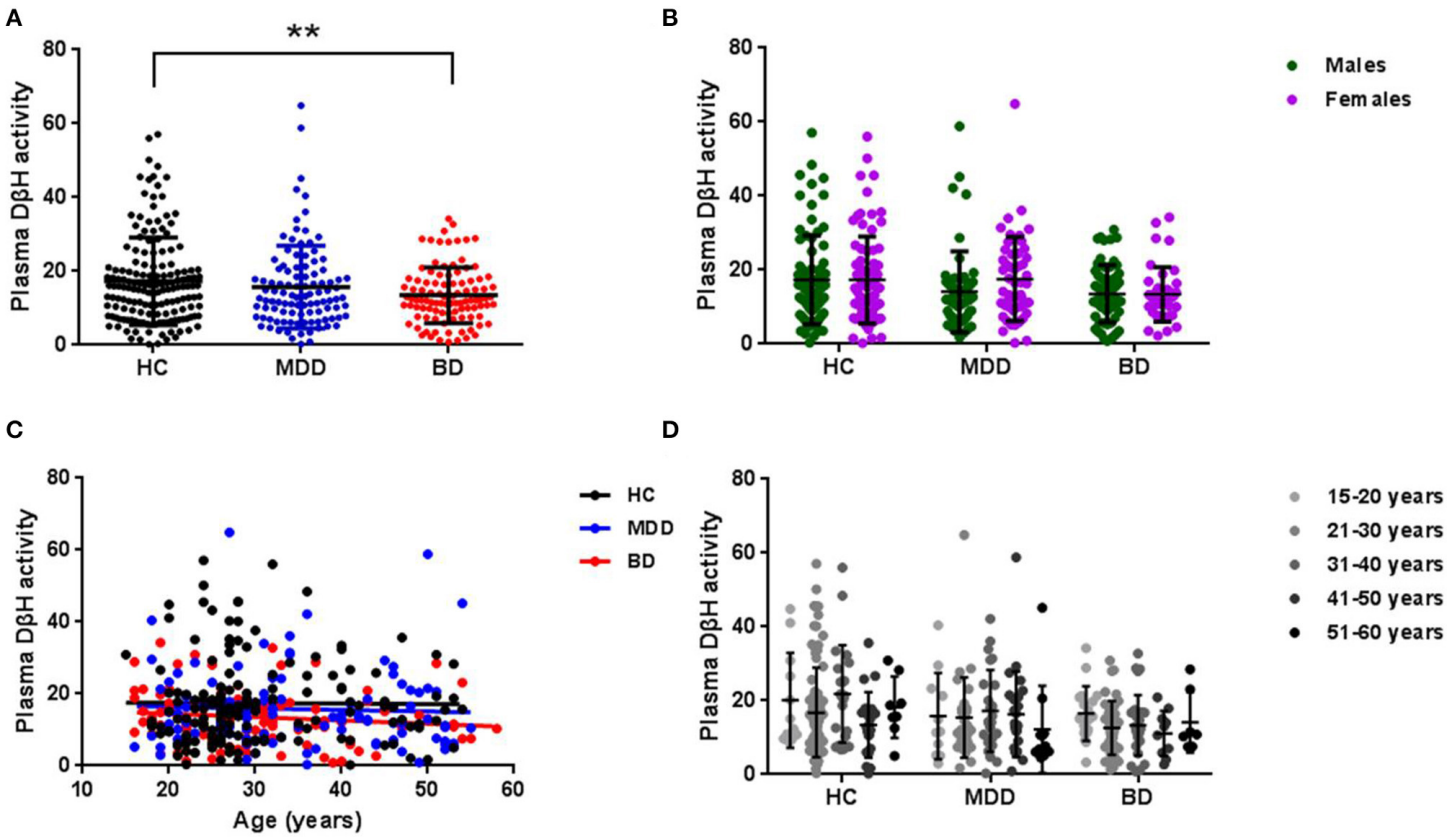
Plasma DβH activity in patients with MDD or BD, and healthy controls (HC). **(A)** Compared the plasma DβH activity in HC (*n* = 160), MDD patients (*n* = 104), and BD patients (*n* = 101). **(B)** Showed the sex difference of DβH activity among HC (*n* = 80 in males; *n* = 80 in females), MDD patients (*n* = 54 in males; *n* = 50 in females), and BD patients (*n* = 62 in males; *n* = 39 in females). **(C,D)** Showed the age-dependent changes of DβH activity in each group. Data was presented as mean ± S.D. **p < 0.01.

The average plasma DβH activity in each group was shown in Figure 1 (the value was 17.31 ± 11.85 in HCs, 15.77 ± 11.19 in MDD patients, 13.49 ± 7.56 in BD patients). Covariance analysis (controlling for age) showed a significant decrease in plasma DβH activity in BD patients compared to HCs (*p* = 0.005). However, there was no significant difference in DβH activity between MDD patients and HCs (*p* = 0.634). It should be noted that no significant difference in plasma DβH activity was found between BD and MDD patients (*p* = 0.245).

In addition, we also analyzed the sex difference in plasma DβH activity among three groups. No significant difference in plasma DβH activity was found between males and females in these three groups (Figure 1B, *F* = 0.830, *p* = 0.363). Figures 1C,D showed there was no significant association in plasma DβH activities with age in each group (all *p* > 0.05).

**Figure 2:**
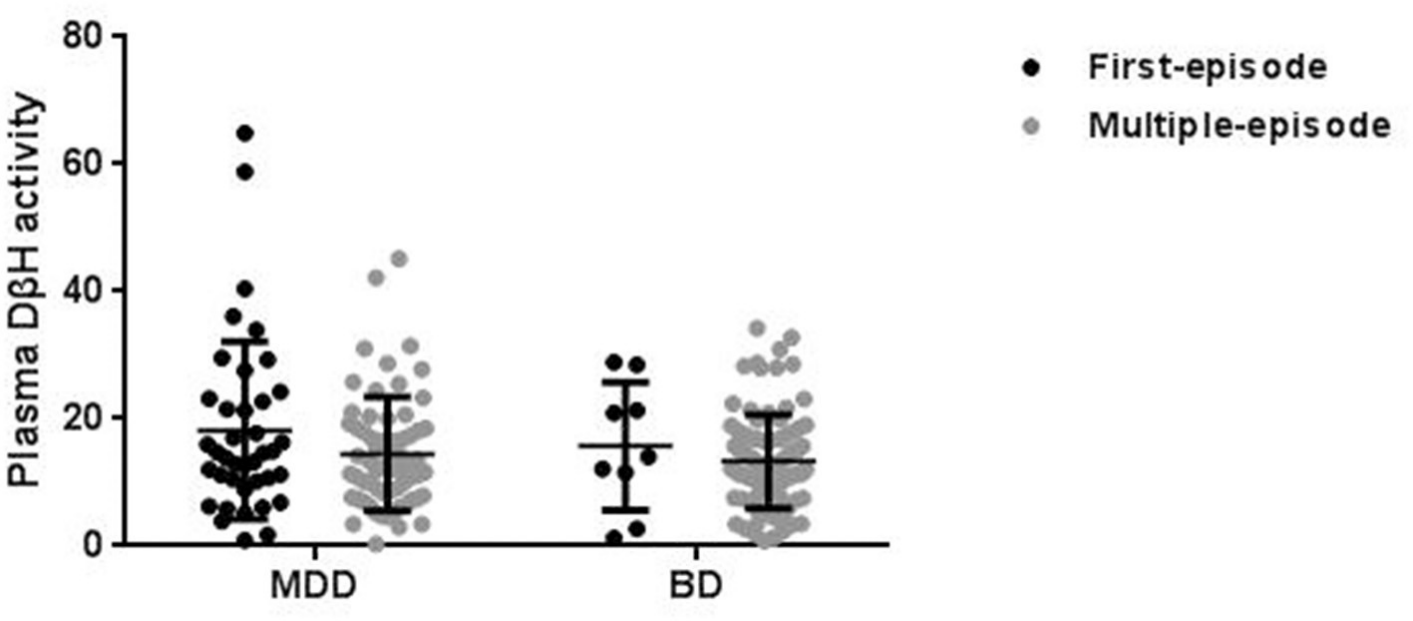
Plasma DβH activity in patients with different episodes, including first-episode (FE) and multi-episode (ME) in MDD and BD. There was no significant difference in DβH activity in patients with different episodes in neither MDD (*n* = 40 in FE; *n* = 64 in ME) nor BD (*n* = 10 in FE; *n* = 91 in ME) groups. Data was presented as mean ± S.D.

In order to exclude confounding factors, the patients were divided into first-episode and multi-episode patients (Figure 2). Only 10% patients (*n* = 10) were in first-episode in BD, while this ratio was 38% (*n* = 40) in MDD. Though there was no significant difference in plasma DβH activity between first-episode and multi-episode patients, the multi-episode patients showed a decrease trend compared with first-episode patients in both MDD (14.45 ± 8.96 and 17.87 ± 13.91, respectively) and BD (13.32 ± 7.35 and 15.10 ± 9.66, respectively).

### Associations of Plasma DβH Activity and Clinical Variables

**Figure 3:**
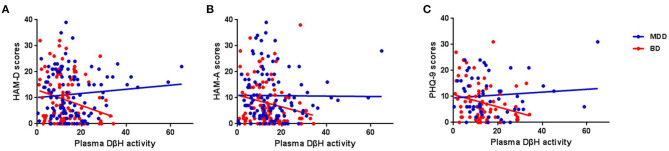
Associations between plasma DβH activity and clinical assessments in MDD and BD patients. **(A–C)** Showed the associations between plasma DβH activity and Hamilton Depression Scale (HAM-D) scores, Hamilton Anxiety Scale (HAM-A) scores, and Patient Health Questionnaire-9 (PHQ-9) scores, respectively. Significant negative correlations were found between DβH activities and HAM-D scores (*r* = −0.234, *p* = 0.021), or HAM-A scores (*r* = −0.201, *p* = 0.041) in BD patients. However, no significant correlation was found between plasma DβH activity and clinical assessments in MDD patients (all *p* > 0.05).

No significant associations were found between DβH activities and clinical assessments or cognitive function in MDD patients (Table 2, all *p* > 0.05). However, significant negative correlations were found between DβH activities and HAM-D scores (*r* = −0.234, *p* = 0.021), or HAM-A scores (*r* = −0.201, *p* = 0.041) in BD patients (Table 3, Figure 3). Nevertheless, plasma DβH activities showed no significant correlation with cognitive assessments in BD patients (all *p* > 0.0167).

**Table 2:** Associations between plasma DβH activity and clinical or cognitive assessments in patients with MDD.

	***N***	**r**	***p***
**Symptom assessment**			
HAM-D	104	0.059	0.559
HAM-A	104	−0.165	0.101
PHQ-9	54	−0.114	0.261
**Cognitive assessment**			
**IQ**	98	−0.083	0.427
**RBANS**	97	−0.066	0.531
**Stroop**			
Single Word Time	93	0.151	0.159
Monochromatic Time	92	−0.085	0.429
Double Words Time	92	0.024	0.822
Double Color Time	91	−0.059	0.588

*MDD, Major depression disorder; HAM-D, Hamilton Depression Scale; HAM-A, Hamilton Anxiety Scale; PHQ-9, Patient Health Questionnaire-9; IQ, Intelligence quotient; RBANS, Repeatable Battery for the Assessment of Neuropsychological Status*.

**Table 3:** Associations between plasma DβH activity and clinical or cognitive assessments in patients with BD.

	***N***	**r**	***p***
**Symptom assessment**			
HAM-D	101	−0.234	0.021
HAM-A	101	−0.201	0.041
PHQ-9	69	−0.105	0.304
**Cognitive assessment**			
**IQ**	97	0.203	0.051
**RBANS**	97	0.103	0.326
**Stroop**			
Single Word Time	94	−0.109	0.308
Monochromatic Time	95	−0.17	0.107
Double Words Time	95	−0.053	0.618
Double Color Time	95	−0.023	0.831

*BD, Bipolar disorder; HAM-D, Hamilton Depression Scale; HAM-A, Hamilton Anxiety Scale; PHQ-9, Patient Health Questionnaire-9; IQ, Intelligence quotient; RBANS, Repeatable Battery for the Assessment of Neuropsychological Status*.

### Discussion

In this study, we enrolled 365 subjects, including 104 MDD patients, 101 BD patients, and 160 HCs, to complete the plasma DβH activity assay and clinical assessments. Five findings were obtained in our study: (1) Patients with BD, not MDD, showed a significant decrease in plasma DβH activities compared with HCs; (2) No significant differences in plasma DβH activities were found between the BD and MDD patients; (3) Significant negative correlations were found between DβH activity and mood-related assessments in BD patients; (4) There was no correlation between DβH activity and cognitive function in BD; (5) In contrast to BD patients, no correlations were found between DβH activity and clinical symptoms or cognition in MDD patients.

In the present study, we found that the plasma DβH activity was significantly lower in BD patients; this was in line with the previous studies in BD (37, 41). It is interesting to note that we also found no significant difference in plasma DβH activity between BD and MDD patients. This may be due to the similarity of biological mechanisms and phenotypes between BD and MDD (30, 33). In addition, the DβH product, NE also showed similar changes in BD and MDD (20, 51–53). For example, NE was used as a stress factor to induced depression in several studies (54, 55), while stress was also a major risk factor of BD (56). Interestingly, there is report showed CSF NE concentration increased while DA levels decreased in rats after stress (57). The decrease of DβH activity may be one of the reasons for the alteration in NE/DA imbalance. The studies of animal models of depression or mania proved the changes in NE system (1, 3, 58–61). Furthermore, the NE metabolism disruption also might be caused by the reduced DβH in BD. The metabolite of NE, MHPG in the circulating system was found higher in BD patients than controls (4, 35), but contrary results were also reported in several studies (10, 20). Interestingly, neuromodulation treatment on mood disorders was also associated with up-regulation of NE system or DβH expression (45, 62, 63). The inconstant results in plasma DβH activity and NE levels in MDD or BD indicated the complex role of DβH in mood disorders. One of the possible reasons for the contrary results might be the difference in detected tissues, such as the blood or the CSF; another reason might be different episodes with the BD patients, such as the manic or the remission state. In addition, the antidepressants, especially NE reuptake inhibitors improved the depressive symptoms directly through regulation of NE levels (64, 65). Together, the present study showed a decrease of plasma DβH activity in BD patients, which may result in the reduced levels of NE to induced the symptoms of patients. Regrettably, plasma levels of NE were not detected in our study.

The main factor affecting DβH activity is heredity. Numerous studies indicate that *DBH* gene is a major quantitative trait locus that regulates blood and CSF DβH activity (25, 66). Previous studies have reported several single nucleotide polymorphisms (SNPs) which correlate with plasma DβH activity (21, 66–68). For example, a previous study reported −1021C>T (rs1611115) accounted for 35–52% of the variation in DβH activity in African American, European American and Japanese (69); while our previous study showed the ratio was 12.6% in Chinese (25). However, few studies explored the association between *DBH* gene polymorphisms and mood disorders. For example, one study showed that the 1603C>T polymorphism of the *DBH* gene is associated with susceptibility to BD in a Turkish population (34). Zhou et al. reported that *DBH* 5′- Ins/Del polymorphism might be associated with susceptibility to MDD in a Chinese population (70). These results indicated the regulated role of *DBH* gene mutation in DβH activity and its association with mood disorders (71).

Though evidence suggested low NE concentration was associated with MDD and BD, several studies showed that NE levels were different in patients between MDD and BD. It is thought that high levels of NE resulted in mania, while low levels of NE led to depression (72). Wiste et al. compared the tyrosine hydroxylase (TH, the key enzyme of DA synthesis) immunoreactive cells in locus coeruleus (LC) among different subjects (73), and found that the TH immunoreactive cells in LC in BD patients were about half of those in controls or MDD patients, suggesting the lower NE transmission in BD. In addition, neuronal damage in LC also emerged in BD, not MDD (74). However, no significant difference in plasma DβH activity was found in our study. This result should be further confirmed due to several confusion factors in the present study. First, only 10% patients (*n* = 10) were in first-episode in BD, while this ratio was 38% (*n* = 40) in MDD in our study. Compared with first-episode patients, multi-episode patients showed decrease trend in both BD and MDD, suggesting the difference in DβH activity might relate to the duration of disease. Second, several studies indicated the influence of antidepressants and mood stabilizers on DβH activity (41, 75–77). In our study, the patients were treated with different psychotropics, including antidepressants (escitalopram, duloxetine), antipsychotics (olanzapine, aripiprazole and quetiapine), and mood stabilizers (lithium and valproic acid). For example, MDD patients were treated with different drugs (76% antidepressants, 19% antipsychotics and 3% mood stabilizers), meanwhile, BD patients were also treated with different kinds of drugs (70% mood stabilizers, 62% antipsychotics, and 27% antidepressants). However, our study did not show the significant difference in patients treated with different kinds of psychotropics (Supplementary Figure 1, *p* > 0.05). Previous studies indicated that the antidepressive effect of mood stabilizers and antidepressants might be partly mediated by DβH and NE system (75, 78). Moreover, the DβH activity was normalized during antidepressant therapy or mood stabilizers treatment (40, 41, 79). However, our present study showed similar plasma DβH activities in patients with different drug treatment groups, suggesting the similar regulation effect of different kinds of drugs. On the other hand, this inconsistency might also relate to the relative small sample size of patients and different medication duration (2–85 months).

To our knowledge, we firstly reported that plasma DβH activity was associated with anxiety and depressive symptoms in BD. Previous studies showed low DβH activity in CSF and serum in patients with mood disorders (37), and lower plasma DβH activity in untreated patients with BD was found than that in controls and lithium-treated patients (41). However, no reports have shown a clear correlation between plasma/serum DβH activity and severity of mood disorders. We found significant negative correlations between plasma DβH activity and anxious and depressive symptoms in BD. In other words, lower DβH activity is associated with more severe anxious or depressive symptoms in BD. This confirmed the important role of plasma DβH activity and monoamine neurotransmitter system in BD. However, no significant correlations were found between plasma DβH activity and mania severity (YMRS scores) in our study. This might relate to the current state of the BD patients when recruited in the present study. About 57% BD patients recruited in our study were in the remission stage, and the others were in the depressive stage (Table 1). It was a great pity that there was no BD patients in the manic state, while the YMRS scores were 3.27 (SD = 6.02). Further study should be conducted in the BD patients with manic state.

However, several limitations should be noted in this study. First, this study focused on the DβH activity in MDD and BD. However, other factors might also affect the dopaminergic transmission, such as polymorphisms of dopaminergic related genes as an endophenotype of MDD. Second, the three study groups, including patients with MDD or BD and HCs, were not fully age-matched. These variables were corrected in the analysis. Third, a majority of patients were receiving antipsychotics and antidepressants, which have confounding effect to explore the role of DβH in mood disorders. Finally, only 10% patients were in first-episode in BD, while the ratio was 38% in MDD.

Taken together, this is a pilot study and shows a reduction of plasma DβH activity as well as hypoactivity of the noradrenergic system in patients with BD. The plasma DβH activity is here proposed as a measure to evaluate the severity of BD.

## Data Availability Statement

The raw data supporting the conclusions of this article will be made available by the authors, without undue reservation.

## Ethics Statement

The studies involving human participants were reviewed and approved by The ethics committee of Beijing Anding Hospital approved the research. Written informed consent to participate in this study was provided by the participants' legal guardian/next of kin.

## Author Contributions

CW, ZS, and QB obtained funding for this study. ZS and QB designed the research. ZS, QB, ZM, FL, WL, FH, and XM performed the experiments and statistical analysis. ZS, QB, YH, and CP wrote the manuscript. All authors contributed to the article and approved the submitted version.

## Conflict of Interest

The authors declare that the research was conducted in the absence of any commercial or financial relationships that could be construed as a potential conflict of interest.

## Supplementary Material

Supplementary Figure 1 Differences of plasma DβH activity in patients treated with different kinds of psychotropics. Data was presented as mean ± S.D.
