# Supplementary figures and images for: Reduced Plasma Dopamine-β-Hydroxylase Activity Is Associated With the Severity of Bipolar Disorder: A Pilot Study
Source: Front Psychiatry. 2021 Apr 28;12:566091. doi: 10.3389/fpsyt.2021.566091 (PMC8115127; doi:10.3389/fpsyt.2021.566091)

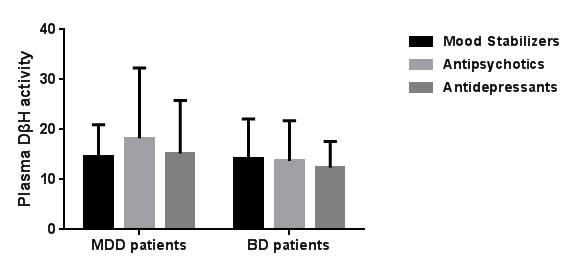

Supplement: Supplementary Figure 1 — Differences of plasma DβH activity in patients treated with different kinds of psychotropics. Data was presented as mean ± S.D. [file Image_1.JPEG]
